# Predictive performance of an antibiotic precision dosing software program in critically ill adults with infection

**DOI:** 10.1093/jac/dkag234

**Published:** 2026-07-24

**Authors:** Paul Williams, Menino Osbert Cotta, Kathryn Wilks, Andras Farkas, Tim Spelman, Jason A Roberts

**Affiliations:** Frazer Institute, Faculty of Health, Medicine and Behavioural Sciences (HMBS), The University of Queensland, Brisbane, Australia; Pharmacy Department, Sunshine Coast University Hospital, 6 Doherty St, Birtinya, Queensland 4575, Australia; Frazer Institute, Faculty of Health, Medicine and Behavioural Sciences (HMBS), The University of Queensland, Brisbane, Australia; Infectious Diseases Department, Sunshine Coast University Hospital, Birtinya, Queensland, Australia; School of Public Health, The University of Queensland, Brisbane, Queensland, Australia; Department of Pharmacy, Cooperman Barnabas Medical Center, 94 Old Short Hills Road, Livingston, NJ 07039, USA; Optimum Dosing Strategies, 63 Reeve Ave, Bloomingdale, NJ 07403, USA; Department of Clinical Neuroscience, Karolinska Institute, Stockholm, Sweden; Department of Health Services Research, Peter MacCallum Cancer Centre, Melbourne, Australia; Centre of Population Health, Burnet Institute, Melbourne, Australia; Clinical Translation Division, Walter & Eliza Hall Institute, Melbourne, Australia; Department of Surgery, St Vincent's Hospital & the University of Melbourne, Melbourne, Australia; School of Medicine, Adelaide University, Adelaide, South Australia, Australia; Frazer Institute, Faculty of Health, Medicine and Behavioural Sciences (HMBS), The University of Queensland, Brisbane, Australia; Herston Infectious Diseases Institute (HeIDI), Metro North Health, Brisbane, Australia; Departments of Pharmacy and Intensive Care Medicine, Royal Brisbane and Women’s Hospital, Brisbane, Australia; UR UM 103, University of Montpellier, Division of Anesthesia Critical Care and Emergency and Pain Medicine, Nimes University Hospital, Nimes, France

## Abstract

**Background:**

Critically ill patients exhibit altered pharmacokinetics, rendering antibiotic dosing challenging. Achieving therapeutic antibiotic exposures may be improved with the use of precision dosing software programs.

**Objectives:**

To quantify and compare both *a priori* and *a posteriori* predictive performance of an antibiotic precision dosing software program in a heterogenous cohort of critically ill adults with infection.

**Methods:**

The precision dosing program, ID-ODS^TM^, was used to predict *a priori* and *a posteriori* concentrations for piperacillin, meropenem, cefepime, flucloxacillin and vancomycin using clinical and demographic data derived from a previous study in critically ill adults (GUIDE trial). Predicted concentrations were compared to observed concentrations using pre-specified acceptance criteria (median predictive error (MDPE) ≤20% and median absolute predictive error (MDAPE) ≤30%), along with F_20_ and F_30_ metrics. Furthermore, the impact of predictions on theoretical dosing recommendations to achieve pre-defined drug exposures was assessed.

**Results:**

The *a priori* predictive performance in 81 patients administered beta-lactams did not meet any pre-specified acceptance criteria (pooled MDPE −35% and MDAPE 58%). However, all beta-lactams met accuracy acceptance criteria for the *a posteriori* approach (pooled MDPE −3.6%), although only cefepime demonstrated acceptable precision and F_20_ and F_30_ acceptance. In 25 patients administered vancomycin, *a priori* predictive performance met all acceptance criteria for accuracy and precision.

When assessing *a priori* theoretical dosing recommendation concordance, approximately one in three predictions led to an unnecessary dosing action. Concordance was similar with an *a posteriori* approach, however, overprediction was observed in 20% of meropenem predictions.

**Conclusions:**

In a heterogenous adult ICU population, the *a priori* predictive performance of vancomycin was shown to be acceptable. Conversely, the *a priori* predictive performance for the beta-lactams was not acceptable. Although predictive performance improved with the *a posteriori* approach for beta-lactams, only cefepime met acceptance criteria.

## Introduction

The rate of serious infections is high among critically ill patients admitted to the ICU. Poor outcomes are often observed in these patients, especially in those with septic shock.^1,2^ Critically ill patients exhibit altered pharmacokinetics (PK) compared with other hospitalized patients, which renders antibiotic dosing challenging.^3,4^ A key objective of antibiotic dosing is to achieve pre-defined PK/pharmacodynamic (PD) targets associated with therapeutic success.^4^ Dosing according to product information in critically ill patients risks sub-therapeutic drug exposure, hence investigations of dosing strategies to optimize therapeutic exposure have been the subject of a growing body of research over the past decade.^5–8^

Therapeutic drug monitoring (TDM) is one strategy that offers an individualized approach to optimize antibiotic therapy. The goal of this approach is to target patient specific antibiotic exposure that give the best probability of treatment success while minimizing the risk of toxicity.^9^ In critically ill patients, a TDM approach has been shown to improve attainment of therapeutic concentrations^5,6,8,10,11^ and is recommended in consensus guidelines.^12–14^

An extension to a traditional TDM approach is to use precision dosing software programs that are informed by population PK models used to predict dosing and/or target attainment. When initiating antibiotic therapy, an *a priori* approach can be used to predict either target drug concentrations or a dosage to achieve a specified PK/PD target and requires input of only basic patient demographic data. An *a posteriori* approach using observed TDM measurements can also be used to predict subsequent antibiotic concentrations. This approach further individualizes dosing and is a key advantage over a traditional TDM approach, in that it can be performed before steady state, allowing for earlier dosing adjustment to attain target PK/PD exposures.^15^ Given that population PK models inform all predictions, and that population samples are derived from specific cohorts of patients, it is imperative that external evaluation of precision dosing software predictive performance is conducted. Previous studies have demonstrated variable predictive performance of model-informed precision dosing across different antibiotics and patient populations, highlighting the importance of external validation in heterogeneous clinical settings where model performance may not be directly transferable.^16–18^

The aim of this *in silico* study was to quantify and compare both *a priori* and *a posteriori* predictive performance of piperacillin, meropenem, cefepime, flucloxacillin and vancomycin in a heterogenous cohort of critically ill adults with infections. Furthermore, we examined the concordance among dosing recommendations to achieve pre-defined PK-PD targets when using predicted versus observed concentrations.

## Materials and methods

### Patient selection and data collection

Clinical characteristics and demographic data, dosing information and plasma drug concentrations from critically ill adults administered piperacillin/tazobactam, meropenem, cefepime, flucloxacillin or vancomycin were obtained from patients enrolled in the GUIDE trial.^19^ The GUIDE trial was a prospective observational study in critically ill adults designed to evaluate the effectiveness of an antibiotic dosing nomogram in achieving therapeutic antibiotic exposures and associated clinical outcomes. While the GUIDE trial included both beta-lactams and vancomycin, the primary publication focused on beta-lactam antibiotics. The present analysis includes patients from this cohort with available TDM data for external model evaluation.

A maximum of four blood samples were taken per patient, with two samples taken during one dosing interval between days 1 and 3 of antibiotic therapy and a further two samples taken during one dosing interval between days 4 and 6 of therapy (if antibiotic therapy was still being administered). Samples taken per dosing interval were one mid-dosing interval sample and one trough sample. Patients were excluded from the present study if an observed trough concentration was unavailable. All other patients formed the external evaluation dataset.

Dosing history and sampling data were collected prospectively, with actual dosing, infusion and sampling times recorded and used for all model evaluations. Where available, administration records in the electronic medical record were reviewed to support consistency of timing data.

### Simulation method to predict antibiotic concentrations and AUC values

ID-ODS^TM^ (Individually Designed Optimum Dosing Strategies, https://www.optimum-dosing-strategies.org/id-ods/) is a simulation tool that supports probabilistic forecasting and Bayesian adaptive feedback powered by the R^®^ software (version 3.6.2; Institute for Statistics and Mathematics, http://www.r-project.org/). ID-ODS was used to perform all dosing simulations using population PK models considered suitable for critically ill patients, which have previously been described in the literature.^20–25^ In our *a priori* approach, patient demographics and dosing information informed the simulation of the predicted trough concentration (or vancomycin AUC_0-24_/MIC) coinciding with the timing of the observed concentration taken (or vancomycin AUC_0-24_/MIC as determined using the trapezoidal method)^26^ between days 1 and 3 of antibiotic therapy. In our *a posteriori* approach, the observed concentrations available from the days 1 to 3 blood samples were used to simulate the predicted trough concentration coinciding with the timing of the observed trough concentration taken between days 4 and 6 of antibiotic therapy.

### Impact of dosing software prediction on theoretical dosing recommendations to achieve pre-defined antibiotic PK/PD targets

To assess how *a priori* and *a posteriori* predictions may influence clinician dosing, concordance among dosing recommendations using predicted versus observed concentrations (or vancomycin AUC_0-24_/MIC) were reported [see Table S1 (available as Supplementary data at *JAC* Online) for details]. ‘Matched’ occurred when the theoretical dosing recommendation resulted in the same action (increased, decreased or unchanged). ‘Underprediction’ occurred when the prediction was less than the observed and an unnecessary dosing recommendation action was required. Examples include a dose increase when the observed concentration would achieve pre-defined PK/PD targets. ‘Overprediction’ occurred when the prediction was greater than the observed, resulting in an unnecessary dosing recommendation. Examples include a dose decrease when the observed concentration would achieve pre-defined PK/PD targets.

### Statistical analysis

Patient demographics and clinical characteristics were presented as either median and IQR or mean and standard deviation depending on the normality of data determined using a Shapiro–Wilk test. Categorical data were presented as number and percentage.

Prediction-based diagnostics were based on the percentage prediction error (PE%) calculated using the equation: PE% = [(predicted − observed)/observed × 100]. Dosing software accuracy was assessed using the median prediction error (MDPE) with a 95% CI. Precision was assessed using the median absolute prediction error (MDAPE) with a 95% CI. Median values were presented as most data were not normally distributed. Accuracy and precision were deemed acceptable if the MDPE ≤±20%, and MDAPE ≤30%, respectively. Composite indices assessing both precision and accuracy were determined using F_20_ (PE% within ±20%) and F_30_ (PE% within ±30%). Acceptance was determined if F_20_ ≥ 35% and F_30_ ≥ 50%. Prediction-based diagnostics were based on the principles of Sheiner and Beal,^27^ and the metrics used in previous studies.^28,29^ Model performance was further evaluated using observed-versus-predicted concentration plots for both *a priori* and *a posteriori* predictions. Each point represents an individual observation–prediction pair. The line of identity (*y* = *x*) was included to facilitate visual assessment of agreement, and to identify potential concentration-dependent bias and variability. In addition, a sub-analysis excluding patients receiving continuous renal replacement therapy (CRRT) was performed. All figures and descriptive analyses were created and performed using GraphPad Prism v.9.3.1.

## Results

In total, the external evaluation dataset included 106 patients (36 prescribed piperacillin/tazobactam, 18 prescribed meropenem, 15 prescribed flucloxacillin, 12 prescribed cefepime and 25 prescribed vancomycin). Table 1 presents patient demographics and clinical characteristics in further detail.

**Table 1.** Patient demographics and clinical characteristics

Characteristic	Piperacillin/tazobactam (*n* = 36)	Meropenem (*n* = 18)	Cefepime (*n* = 12)	Flucloxacillin (*n* = 15)	Vancomycin (*n* = 25)	Combined (*n* = 106)
Age (years)	66.5 (56.5–74.5)	64.3 ± 12.8	67.8 ± 13.1	62.3 ± 13.5	51 (42.5 -63.5)	61.8 ± 13.7
Height (cm)	170.5 ± 10.2	168.9 ± 10.3	170.3 ± 11	172 ± 8.6	175 (166.5–178)	170.8 ± 9.7
Weight (kg)	95.5 (72.5–107.8)	94.9 ± 31.6	91 (71–100)	87.1 (70–98)	82 (67 -110.8)	90 (71.9–108)
BSA (m^2^)	2.09 ± 0.34	2.08 ± 0.37	2.06 (1.82–2.24)	2.05 (1.83–2.12)	2.01 (1.79–2.33)	2.08 ± 0.34
BMI (kg/m^2^)	29.9 (25.7–36.2)	32.28 (24.9–39.1)	29.64 ± 6.43	30.12 (23.03–37.11)	27.7 (23.3–38.9)	29.7 (24.5–36.9)
Male	21 (58.3)	10 (55.5)	7 (58.3)	12 (80)	16 (64)	66 (62.3)
APACHE II score	21 (17–25.5)	19.7 ± 6.1	19 ± 6.4	19.6 ± 5.9	21 (15 -26.5)	21 (15.75–24.25)
SOFA score	6.0 ± 2.8	6.6 ± 3.7	4.9 ± 2.9	5.9 ± 2.4	6.0 ± 2.8	5.9 ± 2.9
Pressors administered on day of first TDM sample	23 (64)	14 (77.8)	7 (58.3)	11 (73.3)	21 (84)	76 (72)
Drains *in situ* on day of first TDM sample	18 (50)	11 (61.1)	5 (41.7)	2 (13.3)	7 (28)	43 (41)
CRRT on day of first TDM sample	8 (22)	5 (27.7)	2 (16.7)	2 (13.3)	6 (24)	23 (22)
eGFR on day 1 of antibiotic therapy	47 (27.9–70.7)	76.7 (43.2 -101.6)	81.8 ± 42.6	58.4 ± 27.3	71.7 (43.9–101.8)	56.5 (36.6–87.3)
Source of infection						
Respiratory	16 (44)	6 (33)	7 (58.3)	3 (20)	11 (44)	43 (41)
Abdominal sepsis	10 (28)	5 (28)	4 (33.3)	1 (6.7)	1 (4)	21 (20)
Skin and soft tissue	3 (8)	2 (11)	0 (0)	7 (46.7)	4 (16)	16 (15)
Urinary sepsis	2 (6)	4 (22)	1 (8.3)	0 (0)	2 (8)	9 (8)
Other	5 (14)	1 (6)	0 (0)	4 (26.7)	7 (28)	17 (16)

Values presented as mean ± SD, median (IQR) or *n* (%). A Shapiro–Wilk test for normality was performed.

BSA, body surface area; BMI, body mass index; APACHE, acute physiology and chronic health evaluation; SOFA, sequential organ failure assessment; eGFR, estimated glomerular filtration rate.

A total of 282 TDM measurements were collected, with 106 observed beta-lactam and vancomycin trough concentrations compared to predicted trough concentrations to measure *a priori* predictability. In addition, 44 observed vancomycin concentrations were used to estimate 22 AUC_0-24_/MIC values and were compared to predicted AUC_0-24_/MIC to assess *a priori* predictability. The median time from antibiotic commencement to the *a priori* evaluation was 33 (23–43.25) hours.

In addition, in 39 patients, 75 observed beta-lactam concentrations collected during one dosing interval between days 4 and 6 of therapy were compared to predicted trough concentrations to measure *a posteriori* predictability. Ninety-five percent of predictions were based on two input TDM samples (a mid-dosing interval and trough sample). The median time from antibiotic commencement to *a posteriori* evaluation was 85 (78 −91) hours. Table S2 presents the number of TDM samples taken according to antibiotic.

**Figure 1.**
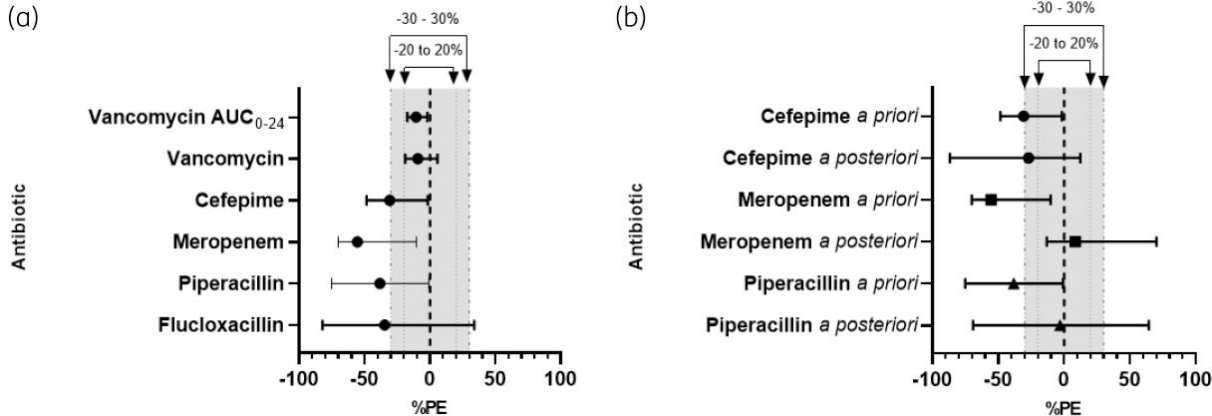
Antibiotic percentage predictive error according to the: (a) *a priori* approach and (b) *a priori* and *a posteriori* (where available). %PE represents percentage predictive error, shapes represent the median value and range represents the interquartile range.

Table 2 presents the accuracy and precision of the *a priori* and *a posteriori* approach according to pooled beta-lactam values and specific antibiotics. Table 3 presents F_20_ and F_30_ acceptance. When assessing the pooled beta-lactam *a priori* approach, the predictive performance did not meet any pre-specified acceptance criteria. However, *a posteriori* accuracy was acceptable for pooled beta-lactams, and precision was improved, although it did not meet acceptance criteria. When assessing specific beta-lactams, no acceptance criteria were met for the *a priori* approach. All beta-lactams met accuracy acceptance criteria for the *a posteriori* approach, however, only cefepime demonstrated acceptable precision and F_20_ and F_30_ acceptance. Meropenem *a posteriori* also met F_20_ acceptance criteria. Accuracy and precision were improved with the *a posteriori* approach for each beta-lactam antibiotic (see Figure 1). Vancomycin *a priori* predictive performance met all acceptance criteria for accuracy and precision.

**Table 2.** *A priori* and *a posteriori* accuracy and precision

Characteristic	Accuracy^a^	CI 95% low	CI 95% high	Acceptance (MDPE ≤ 20%)	Precision^b^	CI 95% low	CI 95% high	Acceptance (MDAPE ≤ 30%)
Pooled beta-lactam *a priori* (*n* = 81)	−35.25	−60.68	−23.64	No	58.29	38.05	66.55	No
Pooled beta-lactam *a posteriori* (*n* = 39)	−3.581	−30.79	17.17	Yes	45.34	17.17	71.68	No
Piperacillin *a priori* (*n* = 36)	−38.31	−75.16	−0.74	No	63.82	38.05	78.92	No
Piperacillin *a posteriori* (*n* = 18)	−3.10	−67.86	45.66	Yes	57.62	17.17	98.02	No
Meropenem *a* priori (*n* = 18)	−55.42	−70.11	−10.24	No	55.94	22.86	70.11	No
Meropenem *a posteriori* (*n* = 15)	8.52	−13.14	70.22	Yes	30.79	8.519	70.22	No
Flucloxacillin *a priori* (*n* = 15)	−34.64	−82.09	33.86	No	69.51	33.86	82.39	No
Cefepime *a priori* (*n* = 12)	−30.79	−48.43	− 1.50	No	31.71	20.53	48.43	No
Cefepime *a posteriori* (*n* = 6)	−26.99	−86.69	12.48	Yes	28.43	0.023	86.69	Yes
Vancomycin *a priori* (*n* = 25)	−9.33	−19.00	5.79	Yes	22.78	13.08	30.27	Yes
Vancomycin AUC_0-24_ *a priori* (*n* = 22)	−10.63	−17.24	−1.96	Yes	15.54	10.63	27.62	Yes

^a^According to MDPE.

^b^According to MDAPE.

**Table 3.** F_20_ and F_30_ acceptance criteria

Characteristics	F_20_^a^, *n* (%)	Acceptance (F_20_ ≥ 35%)	F_30_^b^, *n* (%)	Acceptance (F_30_ ≥ 50%)
Pooled beta-lactam *a priori* (*n* = 81)	13 (16)	No	23 (28)	No
Pooled beta-lactam *a posteriori* (*n* = 39)	13 (33.3)	No	15 (38.4)	No
Piperacillin *a priori* (*n* = 36)	6 (16.7)	No	9 (25)	No
Piperacillin *a posteriori* (*n* = 18)	5 (27.7)	No	5 (27.7)	No
Meropenem *a priori* (*n* = 18)	3 (16.7)	No	7 (38.9)	No
Meropenem *a posteriori* (*n* = 15)	5 (33.3)	Yes	7 (46.7)	No
Flucloxacillin *a priori* (*n* = 15*)*	2 (13.3)	No	2 (13.3)	No
Cefepime *a priori* (*n* = 12)	2 (16.7)	No	5 (41.7)	No
Cefepime *a posteriori* (*n* = 6)	3 (50)	Yes	5 (50)	Yes
Vancomycin *a priori* (*n* = 25)	12 (48)	Yes	17 (68)	Yes
Vancomycin AUC_0-24_ *a priori* (*n* = 22)	14 (66.7)	Yes	16 (76.2)	Yes

^a^Calculated as predictive error percentage with ±20% (measure of precision and accuracy).

^b^Calculated as predictive error percentage with ±30% (measure of precision and accuracy).

Observed-versus-predicted plots are presented in Figure S1 and demonstrated substantial scatter around the line of identity, consistent with the observed imprecision. *A priori* beta-lactam predictions showed a predominance of underprediction, with improved alignment observed following *a posteriori* estimation, although variability remained, with occasional deviations above the line of identity. By contrast, vancomycin predictions more closely aligned with the line of identity.

In our sub-analysis that excluded those receiving CRRT, acceptance rates remained largely the same, however, cefepime predictive performance worsened (see Tables S3 and S4). Data were not available to assess vancomycin and flucloxacillin *a posteriori* predictive performance.

**Figure 2.**
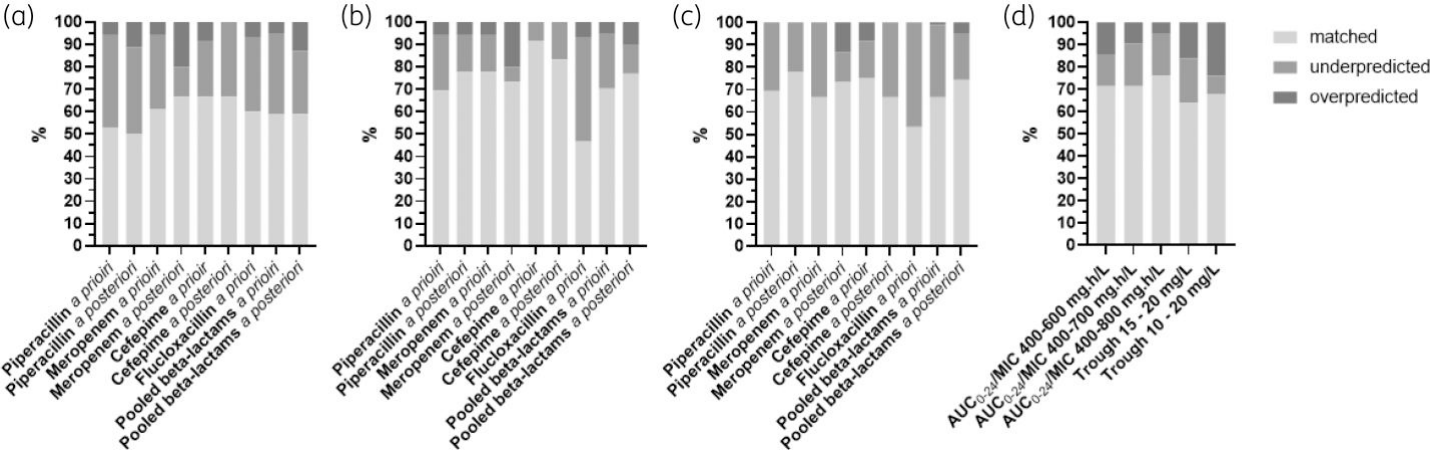
Impact of predictions on dosing recommendations to achieve (a) 100% fT1-4xMIC, (b) 100% fT1-10xMIC, (c) 100% fT4-10xMIC and (d) various vancomycin PK/PD targets. 100% fT(*X*) × MIC denotes 100% of the time in which the unbound concentration remained above *X* times the minimum inhibitory concentration as a percentage of the dosing interval.

When assessing *a priori* theoretical dosing recommendation concordance to achieve 100% *f*T 1-4 MIC, our results showed that 53%–67% of beta-lactam predictions matched and 25%–42% underpredicted (see Figure 2). Concordance was similar with an *a posteriori* approach, however, overprediction was observed in 20% of meropenem predictions. Vancomycin *a priori* predictions performed better, with 71% matching when targeting an area under the curve (AUC) _0-24_/MIC 400-700 mg.h/L, however, 19% underpredicted (see Figure 2).

## Discussion

To the best of our knowledge, we present the broadest predictive performance evaluation of a range of antibiotics included in a single dosing software program assessed in a heterogenous cohort of critically ill patients with serious infection. The predictive performance of vancomycin was the only antibiotic to meet all acceptance criteria for the *a priori* approach. Although, an *a posteriori* approach largely improved predictive performance, cefepime was the only one to meet all acceptance criteria, and all other beta-lactams demonstrated acceptable accuracy only. Approximately 1 in 3 predictions led to an unnecessary dosing action according to the theoretical dose action approach. These findings were supported by observed-versus-predicted plots, which demonstrated substantial variability in beta-lactam predictions, with some deviation from the line of identity at higher observed concentrations, and closer agreement for vancomycin, although these observations were based on visual assessment.

Our vancomycin *a priori* results align with a recent study where vancomycin predictions were shown to be more accurate and precise as compared to piperacillin and meropenem in a heterogenous cohort of critically ill patients.^16^ Furthermore, in a 2019 study, Broeker *et al*. determined the Goti model (also used in our evaluation) the most suitable model for use in hospitalized patients.^17^

Piperacillin predictive performance was shown to be imprecise in both our *a priori* and *a posteriori* evaluation, however, *a posteriori* had acceptable accuracy. The Felton *et al*. model^21^ has been externally evaluated in a similar cohort previously.^16^ Our results align with their findings, however, acceptance criteria were not applied. Differences in severity of illness and infection profiles between our patients and the population PK sample may account for the discordance observed in our results. In addition, patients included in our study weighed more and had worse renal function. External evaluation mismatch has previously been shown to negatively affect prediction performance.^17^

Meropenem *a priori* predictions were the least accurate in our evaluation. Our findings aligns with Chai *et al.*’s evaluation of the Crandon model (also used in our evaluation) according to their percentage bias results.^16^ Meropenem was found to be imprecise in both ours and a previous evaluation of the same model.^18^ Our study, which included patients with various serious infections and higher illness severity scores, differed from the Crandon population sample that mostly included patients with ventilator-associated pneumonia. These differences may have contributed to discordance observed in our findings.

Among all PK models assessed, the cefepime model was the closest match to our study population in terms of illness severity score, estimated renal function and infection profile.^22^ Although the *a priori* approach for cefepime did not meet any acceptance criteria, the *a posteriori* approach did meet all acceptance criteria, albeit with a small sample size. A larger external evaluation is required to confirm our acceptance results, especially given that acceptance failed when excluding patients with CRRT.

In our evaluation, the *a priori* predictive performance of flucloxacillin was inaccurate and the most imprecise. Interestingly, the clinical characteristics were well matched to the population PK sample developed by Jager *et al*.^25^ Our results were similar to one of two external evaluation cohorts conducted by Jager *et al*., while performance was vastly improved in their other cohort. This highlights the variability in predictive performance across different patient cohorts, suggesting a need for larger external evaluations for more definitive results.

Our evaluation demonstrated that the *a priori* approach often underpredicted observed antibiotic concentrations, most notably with beta-lactams. This finding aligns with the findings of Chai *et al.*, where the frequency of underprediction was ∼40%.^16^ It is concerning that ∼25%–40% of predictions would have led to an unnecessary dosing recommendation to achieve the pre-defined PK/PD target. This underprediction was improved with the *a posteriori* approach, which may reduce the need for further TDM in certain situations (e.g. favourable clinical progression), due to clinicians being confident that adequate drug exposure has been attained. However, our study found that the *a posteriori* approach tended to overpredict in the case of meropenem, which could inadvertently result in underdosing in clinical practice.

The performance of *a posteriori* predictions is influenced by both the number and timing of concentration measurements available for Bayesian updating. While additional early samples may improve the reliability of pharmacokinetic parameter individualization, this must be balanced against the practical constraints of increased sampling, including patient burden, laboratory capacity and clinical workflow. Future studies incorporating optimal design approaches may help define sparse sampling strategies (e.g. trough-only versus combinations of mid-interval and trough concentrations) that optimize predictive performance while maintaining feasibility within routine clinical practice.

### Limitations

The findings of our study should be balanced against the notably limitations. First, there was variability in the timing of TDM measurements. Given the known PK variability in our cohort, it is possible that clinical characteristics changed during this time, that may have affected the predictive performance. However, we feel the timing of samples was a reasonable reflection of clinical practice but recognize that standardized timing of TDM measurements could have improved the methodology. Second, sampling errors in the prospective data collection cannot be ruled out, and inaccurate administration records in the electronic medical record may be possible. Such errors would have led to inaccurate evaluation results, however, multiple visual and verbal reminders were given to mitigate these risks.

Third, most *a posteriori* evaluations were based on two TDM measurements, therefore, results may be more favourable than expected from a single TDM sample, which is more likely to occur in clinical practice. While additional samples may improve the reliability of Bayesian parameter estimation, this must be balanced against the practical constraints of increased sampling. However, as most *a posteriori* results did not meet acceptance criteria, the overall interpretation is unlikely to differ substantially between one and two TDM samples.

Fourth, interpretation of observed-versus-predicted plots was based on visual assessment and limited by sample size, particularly for individual antibiotics and *a posteriori* analyses. As such, these findings should be considered exploratory and were not formally evaluated.

Fifth, this study evaluated predictive performance within a single dosing software platform. As such, direct comparisons with other precision dosing tools were not performed, which limits the ability to contextualize these findings relative to alternative software platforms.

Finally, the theoretical dosing action analysis does not capture the magnitude of dose change required. For example, a predicted and observed concentration of 8 and 30 mg/L would be considered ‘matched’ for the cefepime PK/PD target of 1–4× MIC. In clinical practice, however, the subsequent dosing action in this scenario may not necessarily be the same.

### Conclusions

Despite these limitations, in a heterogenous adult ICU population, the *a priori* predictive performance of vancomycin was shown to be acceptable, supporting further evaluation of patient-centric outcomes. Conversely, the *a priori* predictive performance for the beta-lactam antibiotics was not acceptable. However, predictive performance improved with the *a posteriori* approach, only cefepime met acceptance criteria. We believe the likelihood of unnecessary dosing occurring following predictions is not currently clinically acceptable. Further evaluation is required, and perhaps specific population PK models designed to inform dosing software programs are needed to improve on the predictive performance capabilities of dosing software programs.

## Supplementary Material

dkag234_Supplementary_Data

## Supplementary data

Figure S1 and Tables S1–S4 are available as Supplementary data at JAC Online.
