# Supplementary material for: Predictive performance of an antibiotic precision dosing software program in critically ill adults with infection
Source: J Antimicrob Chemother. 2026 Jul 24;81(8):dkag234. doi: 10.1093/jac/dkag234 (PMC13399157; doi:10.1093/jac/dkag234)
Supplement: dkag234_Supplementary_Data [file dkag234_supplementary_data.docx]

## Supplementary material

Table S1: PK/PD targets and MIC values

| **PK/PD targets** | |
| --- | --- |
| **Beta-lactams^*^:**   - Unbound concentration 1–4 times the MIC (100%*f*T>1-4XMIC) - Unbound concentration 1–10 times the MIC (100%*f*T>1-10XMIC) - Unbound concentration 4–10 times the MIC for the (100%*f*T> 4-10XMIC)   ^*^Targets based on efficacy exposures while not exceeding toxicity exposures. (1)  **Vancomycin^*^:**   - area under the 24 h total concentration-time curve to minimum inhibitory concentration ratio (AUC_0-24_/MIC) of 400 – 600 mg.h/L - AUC_0-24_/MIC 400 – 700 mg.h/L - AUC_0-24_/MIC 400 – 800 mg.h/L - Trough total concentration 15 – 20 mg/L - Trough total concentration 10 – 20 mg/L   ^*^Targets based on various efficacy exposures while minimising toxicity exposures. (1-3) | |
| **Beta-lactams**: the epidemiological cut-off (ECOFF) values for *Pseudomonas aeruginosa*, determined by the European Committee on Antimicrobial Susceptibility Testing (EUCAST) were used to determine the highest MICs expected from wild-type populations (those without acquired or mutational resistance). (4) These ECOFF MIC values correlate to the current EUCAST clinical breakpoints (Version 12.0), (5) for “resistant” (a high likelihood of therapeutic failure even when there is increased exposure to the antimicrobial agent). EUCAST and the Clinical and Laboratory Standards Institute (CLSI) do not publish ECOFF values or clinical breakpoints for *Staphylococcus aureus* and flucloxacillin. A surrogate MIC of 2 mg/L, derived from oxacillin, (6) a similarly active isoxazole penicillin, (7) was used. | |
| **Vancomycin:** Although the EUCAST clinical breakpoint (Version 12.0) for MRSA is 2 mg/L, (5) an alternative agent should be considered when the MIC is >1 due to the low probability of achieving therapeutic exposure without heightening risk of toxicity; (2) hence a surrogate MIC of 1 mg/L was used. | |
| **Antibiotic** | **MIC values (mg/L)** |
| Piperacillin | 16 |
| Meropenem | 2 |
| Flucloxacillin | 2 |
| Cefepime | 8 |
| Vancomycin | 1 |

Table S2: Number of TDM samples according to antibiotic and timing of sample

| Antibiotic | Days 1-3 mid-sample | Days 1-3 trough | Days 4-6 mid-sample | Days 4-6 trough | Total |
| --- | --- | --- | --- | --- | --- |
| Piperacillin | 36 | 36 | 17 | 18 | 107 |
| Meropenem | 17 | 18 | 13 | 15 | 63 |
| Flucloxacillin | 14 | 15 | 0 | 0 | 27 |
| Cefepime | 12 | 12 | 6 | 6 | 36 |
| Vancomycin | 22 | 25 | 0 | 0 | 47 |
| Total | 101 | 106 | 36 | 39 | 282 |

*Figure S1: Observed versus predicted antibiotic concentrations. Each point represents an individual observation-prediction pair. The dashed line represents the line of identity (y = x).*


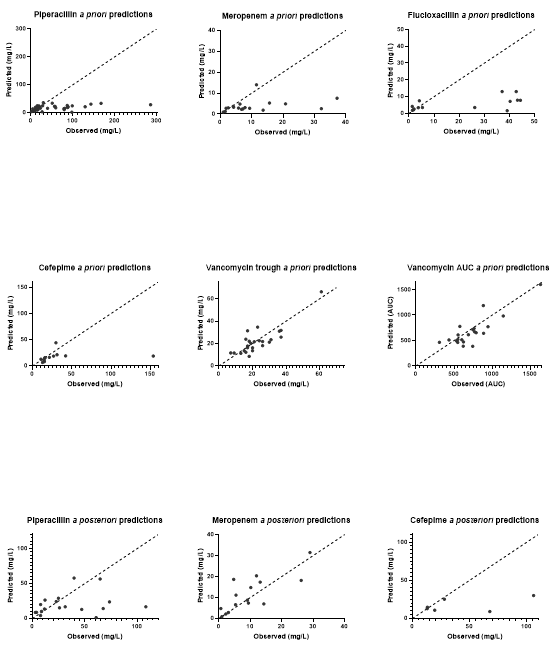


Table S3: A priori and a posteriori accuracy and precision (excluding CRRT)

| Characteristic | Accuracy* | CI 95% low | CI  95% high | Acceptance (MDPE ≤ 20%) | Precision^¥^ | CI 95% low | CI 95% high | Acceptance (MDAPE ≤ 30%) |
| --- | --- | --- | --- | --- | --- | --- | --- | --- |
| Pooled *a priori* (n = 83) | -23.75 | -34.64 | -10.24 | No | 38.05 | 29.89 | 52.78 | No |
| Pooled beta-lactam *a priori*  (n = 64) | -33.76 | -59.92 | -11.07 | No | 49.29 | 34.64 | 66.55 | No |
| Pooled beta-lactam  *a posteriori (n = 15)* | 0.02 | -44.38 | 23.41 | Yes | 47.38 | 23.41 | 79.09 | No |
| Piperacillin *a priori (n = 28)* | -34 | -70 | 9.02 | No | 60.21 | 35.25 | 78.51 | No |
| Piperacillin  *a posteriori (n = 15)* | 16.98 | -67.86 | 120.4 | Yes | 67.86 | 40.87 | 120.4 | No |
| Meropenem *a priori (n =13)* | -50.16 | -76.4 | -10.24 | No | 50.16 | 18.3 | 76.4 | No |
| Meropenem  *a posteriori (n = 11)* | 8.52 | -30.79 | 298 | Yes | 30.79 | 6.229 | 298 | No |
| Flucloxacillin *a priori (n = 13)* | -34.64 | -82.09 | 33.86 | No | 64.89 | 32.94 | 82.09 | No |
| Cefepime *a priori (n =10)* | -30.79 | -54.64 | 20.53 | No | 38.95 | 20.53 | 54.64 | No |
| Cefepime *a posteriori (n = 5)* | -44.38 | -86.69 | 12.48 | No | 44.38 | 0.02 | 86.69 | No |
| Vancomycin *a priori (n = 19)* | -9.33 | -23.75 | 22.78 | Yes | 22.78 | 9.33 | 30.38 | Yes |
| Vancomycin AUC_0-24_  *a priori (n = 16)* | -4.80 | -24.24 | 18.29 | Yes | 17.76 | 4.918 | 35.3 | Yes |

*Abbreviations: CRRT, continuous renal replacement therapy; CI, confidence interval; * according to median prediction error (MDPE); ¥ according to median absolute prediction error (MDAPE); n, number; AUC, Area Under the Curve.*

Table S4: F_20_ and F_30_ acceptance criteria (excluding CRRT)

| Characteristics | F_20_ ^¥^_,_  n (%) | Acceptance  (F_20_ ≥ 35%) | F_30_^*^, n (%) | Acceptance  (F_30_ ≥ 50%) |
| --- | --- | --- | --- | --- |
| Pooled *a priori* (n = 83) | 20 (24.1) | No | 33 (39.76) | No |
| Pooled beta-lactam *a priori* (n = 64) | 11 (17.19) | No | 19 (29.69) | No |
| Pooled beta-lactam *a posteriori* (n = 31) | 9 (29.03) | No | 10 (32.26) | No |
| Piperacillin *a priori* (n = 28) | 5 (17.9) | No | 7 (25) | No |
| Piperacillin *a posteriori* (n = 15) | 3 (20) | No | 3 (20) | No |
| Meropenem *a priori* (n = 13) | 3 (23.08) | No | 6 (46.15) | No |
| Meropenem *a posteriori* (n = 11) | 4 (36.36) | Yes | 5 (45.45) | No |
| Flucloxacillin *a priori* (n = 13) | 2 (15.38) | No | 2 (15.38) | No |
| Cefepime *a priori* (n = 10) | 1 (10) | No | 1 (10) | No |
| Cefepime *a posteriori* (n = 5) | 2 (40) | Yes | 2 (40) | No |
| Vancomycin *a priori* (n = 19) | 9 (47.4) | Yes | 14 (73.7) | Yes |
| Vancomycin AUC_0-24_ *a priori* (n = 16) | 9 (56.25) | Yes | 11 (68.75) | Yes |

*Abbreviations: CRRT, continuous renal replacement therapy; ¥ calculated as predictive error percentage with +/- 20% (measure of precision and accuracy); ^*^ calculated as predictive error percentage with +/- 30% (measure of precision and accuracy); n, number; AUC, Area Under the Curve.*

## References

1. Abdul-Aziz MH, Alffenaar J-WC, Bassetti M, Bracht H, Dimopoulos G, Marriott D, et al. Antimicrobial therapeutic drug monitoring in critically ill adult patients: a Position Paper#. Intensive Care Medicine. 2020;46(6):1127-53.

2. Rybak MJ, Le J, Lodise TP, Levine DP, Bradley JS, Liu C, et al. Therapeutic monitoring of vancomycin for serious methicillin-resistant Staphylococcus aureus infections: A revised consensus guideline and review by the American Society of Health-System Pharmacists, the Infectious Diseases Society of America, the Pediatric Infectious Diseases Society, and the Society of Infectious Diseases Pharmacists. American Journal of Health-System Pharmacy. 2020;77(11):835-64.

3. Rybak M, Lomaestro B, Rotschafer JC, Moellering R, Jr., Craig W, Billeter M, et al. Therapeutic monitoring of vancomycin in adult patients: a consensus review of the American Society of Health-System Pharmacists, the Infectious Diseases Society of America, and the Society of Infectious Diseases Pharmacists. American journal of health-system pharmacy : AJHP : official journal of the American Society of Health-System Pharmacists. 2009;66(1):82-98.

4. European Committee on Antimicrobial Suseptibilty Testing. Data from the EUCAST MIC distribution website, last accessed 16th May 2022”. <http://www.eucast.org>"

5. European Committee on Antimicrobial Suseptibilty Testing. Clinical Breakpoint Tables, Dosages, version 12.0, 2022 [Internet]. [cited 16/05/2022]. Available from: <http://www.eucast.org/clinical_breakpoints/>.

6. Weinstein MP. Performance standards for antimicrobial susceptibility testing : supplement M100. 31st edition. ed. Clinical, Laboratory Standards I, editors. Wayne, Pa.: Clinical and Laboratory Standards Institute; 2021.

7. Sutherland R, Croydon EAP, Rolinson GN. Flucloxacillin, a New Isoxazolyl Penicillin, Compared with Oxacillin, Cloxacillin, and Dicloxacillin. Br Med J. 1970;4(5733):455-60.
